# Supplementary material for: YBEY is an essential biogenesis factor for mitochondrial ribosomes
Source: Nucleic Acids Res. 2020 Mar 17;48(17):9762–86. doi: 10.1093/nar/gkaa148 (PMC7515705; doi:10.1093/nar/gkaa148)

SUPPLEMENTARY DATA

for

YBEY is an essential biogenesis factor for mitochondrial ribosomes

Sabrina Summer, Anna Smirnova, Alessandro Gabriele, Ursula Toth, Fasemore Mandela, Konrad U. Förstner, Lauriane Kuhn, Johana Chicher, Philippe Hammann, Goran Mitulović, Nina Entelis, Ivan Tarassov, Walter Rossmanith and Alexandre Smirnov

SUPPLEMENTARY TABLES

Table S1. *E. coli* strains used in this study.

Strain ID	Background	Plasmids	Resistance	Overexpressed protein gene
SAB0021	BL21 Star(DE3)	pSAP0007	Amp^R	*His*$_6$*-MRPL18*
SAB0103	Rosetta	pSAP0077	Amp^R	*YBEY-His*$_6$
SAB0116	Rosetta	pSAP0079	Amp^R	$YBEY^{R55A}$*-His*$_6$
SAB0117	Rosetta	pSAP0080	Amp^R	$YBEY^{R57A}$*-His*$_6$
SAB0118	Rosetta	pSAP0081	Amp^R	$YBEY^{D62A}$*-His*$_6$
SAB0119	Rosetta	pSAP0082	Amp^R	$YBEY^{H118A}$*-His*$_6$
SAB0120	Rosetta	pSAP0083	Amp^R	$YBEY^{H122A}$*-His*$_6$
SAB0121	Rosetta	pSAP0084	Amp^R	$YBEY^{H128A}$*-His*$_6$
SAB0122	Rosetta	pSAP0085	Amp^R	$YBEY^{M137A}$*-His*$_6$
SAB0123	Rosetta	pSAP0086	Amp^R	$YBEY^{E141A}$*-His*$_6$
SAB0144	Rosetta	pSAP0097	Kan^R	*C1QBP*
SAB0146	Rosetta	pSAP0077, pSAP0097	Amp^R, Kan^R	*YBEY-His*$_6$*, C1QBP*
SAB0199	Rosetta	pSAP0112	Kan^R	*MRPS11*

Table S2. Human cell lines used in this study.

Cell line	Background	Genotype
Flp-In T-REx 293	Flp-In T-REx 293	WT
SAL001	Flp-In T-REx 293	*FRT::Hyg::YBEY-3×FLAG::FRT::lacZ-Zeo*
HepG2	HepG2	WT
HeLa	HeLa	WT
293T-REx	293T-REx	WT ($YBEY^+$)
$YBEY^+$_clone1	293T-REx	$YBEY^+$
$YBEY^+$_clone2	293T-REx	$YBEY^+$
$YBEY^+$_clone3	293T-REx	$YBEY^+$
YBEY KO1	293T-REx	Δ*YBEY*
YBEY KO2	293T-REx	Δ*YBEY*
YBEY KO3	293T-REx	Δ*YBEY*
YBEY KO2-YBEY_clone1	293T-REx	Δ*YBEY(KO2)::YBEY*
YBEY KO2-YBEY_clone2	293T-REx	Δ*YBEY(KO2)::YBEY*
YBEY KO2-YBEY-R55A_clone1	293T-REx	Δ*YBEY(KO2)::*$YBEY^{R55A}$
YBEY KO2-YBEY-R55A_clone2	293T-REx	Δ*YBEY(KO2)::*$YBEY^{R55A}$
YBEY KO2-YBEY-H128A_clone1	293T-REx	Δ*YBEY(KO2)::*$YBEY^{H128A}$
YBEY KO2-YBEY-H128A_clone2	293T-REx	Δ*YBEY(KO2)::*$YBEY^{H128A}$
YBEY KO3-YBEY_clone1	293T-REx	Δ*YBEY(KO3)::YBEY*

YBEY KO3-YBEY_clone2	293T-REx	Δ*YBEY(KO3)::YBEY*
YBEY KO3-YBEY-R55A_clone1	293T-REx	Δ*YBEY(KO3)::YBEY*^{R55A}
YBEY KO3-YBEY-R55A_clone2	293T-REx	Δ*YBEY(KO3)::YBEY*^{R55A}
YBEY KO3-YBEY-H128A_clone1	293T-REx	Δ*YBEY(KO3)::YBEY*^{H128A}
YBEY KO3-YBEY-H128A_clone2	293T-REx	Δ*YBEY(KO3)::YBEY*^{H128A}

Table S3. Oligonucleotides used in this study.

Table S4. Antibodies used in this study.

**Antibody**	**Manufacturer**	**Reference**
Goat polyclonal anti-Actin (C-11)	Santa Cruz Biotechnology	sc-1615
Goat polyclonal anti-ALDH2 (N-14)	Santa Cruz Biotechnology	sc-48838
Mouse monoclonal anti-ATP5A (clone 7H10)	Molecular Probes	A-21350
Mouse monoclonal anti-CYC1(clone 16D10)	Molecular Probes	A-21362
Mouse monoclonal anti-COX1 (clone 1D6E1A8)	Abcam	ab14705
Mouse monoclonal anti-COX2 (clone 12C4)	Molecular Probes	A-6404
Mouse monoclonal anti-COX4 (clone 20E8)	Santa Cruz Biotechnology	sc-58348
Mouse monoclonal anti-cytochrome c (clone 6H2.B4 (RUO))	BD Biosciences	556432
Mouse monoclonal anti-FLAG (clone M2)	Sigma-Aldrich	F1804
Mouse monoclonal anti-GAPDH (clone 6C5)	Millipore	MAB374
Mouse monoclonal anti-HDAC2 (clone 3F3)	Santa Cruz Biotechnology	sc-81599
Mouse monoclonal anti-p32 (clone H-9)	Santa Cruz Biotechnology	sc-271200
Mouse monoclonal anti-SDHA (clone 2E3)	Molecular Probes	A-11142
Mouse monoclonal anti-VDAC1 (clone B-6)	Santa Cruz Biotechnology	sc-390996
Rabbit polyclonal anti-bL20m	Sigma-Aldrich	HPA047074
Rabbit polyclonal anti-ERAL1	Sigma-Aldrich	HPA021425
Rabbit polyclonal anti-HA	Sigma-Aldrich	H6908
Rabbit polyclonal anti-mL38	This manuscript	3528
Rabbit polyclonal anti-mS27	Sigma-Aldrich	HPA071751
Rabbit polyclonal anti-mS35	Proteintech Group	16457-1-AP
Rabbit polyclonal anti-mS37	Proteintech Group	11728-1-AP
Rabbit polyclonal anti-mtIF3	Proteintech Group	14219-1-AP
Rabbit polyclonal anti-NDUFB8	Sigma-Aldrich	HPA003886
Rabbit polyclonal anti-OPA1	Proteintech Group	27733-1-AP
Rabbit polyclonal anti-PNPase	Abcam	ab96176
Rabbit polyclonal anti-TOMM20 (FL-145)	Santa Cruz Biotechnology	sc-11415

Rabbit polyclonal anti-uL22m	Proteintech Group	16299-1-AP
Rabbit polyclonal anti-uL24m	Proteintech Group	16224-1-AP
Rabbit polyclonal anti-uL4m	Proteintech Group	27484-1-AP
Rabbit polyclonal anti-uS11m	Proteintech Group	17041-1-AP
Rabbit polyclonal anti-YBEY	Sigma-Aldrich	HPA018162
Donkey polyclonal anti-Rabbit IgG	Sigma-Aldrich	NA934V
Goat polyclonal anti-Rabbit IgG	Agilent	P0448
Goat polyclonal anti-Rabbit IgG	Cell Signaling Technology	7074
Mouse m-IgGκ BP-HRP	Santa Cruz Biotechnology	sc-516102
Rabbit polyclonal anti-Goat IgG	Sigma-Aldrich	A8919
Rabbit polyclonal anti-Mouse IgG	Agilent	P0260
Sheep polyclonal anti-Mouse IgG	GE Healthcare	NXA931
Goat polyclonal anti-Mouse IgG-Alexa Fluor 488	Thermo Fisher Scientific	A-11001
Goat polyclonal anti-Mouse IgG-Alexa Fluor 555	Thermo Fisher Scientific	A32727
Goat polyclonal anti-Mouse IgG-Alexa Fluor 647	Thermo Fisher Scientific	A-21237
Goat polyclonal anti-Rabbit IgG-Alexa Fluor 488	Thermo Fisher Scientific	A-11008
Goat polyclonal anti-Rabbit IgG-Alexa Fluor 555	Thermo Fisher Scientific	A-21429
Goat polyclonal anti-Rabbit IgG-Alexa Fluor 647	Thermo Fisher Scientific	A-21246

Table S5. Differential gene expression analysis of the mitochondrial transcriptomes of *YBEY*$^{+}$ and *YBEY* KO cells. Mitochondrial RNAs from three independent cell lines for each genotype were sequenced and the data analysed by DESeq2.

Table S6. Proteins identified by LC-MS/MS that reproducibly copurified with YBEY-3×FLAG from mitochondrial lysates of HEK293T-REx cells, as compared to the untagged parental control cell line. Mitocarta2.0 proteins are shown in bold.

Table S7. Differential quantitative MS analysis of mitoribosomal and mitoribosome-associated/copurifying proteins from three independent *YBEY*$^{+}$ and *YBEY* KO cell lines. "INF" in the "Fold change" column means an infinite enrichment due to the absence of the corresponding peptides in any of the *YBEY*$^{+}$ replicates. Mitoribosomal and mitoribosome-associated proteins are bold, those significantly underrepresented in *YBEY* KO (P_{adj} < 0.05) are highlighted with yellow, and those depleted >10-fold are red. Weighted spectral counts from three biological replicates per genotype (quantified in 2-3 technical runs per replicate) are also provided.

SUPPLEMENTARY FIGURE LEGENDS

Figure S1. Occurrence of *YBEY* genes in bacterial and eukaryal clades. Phylogenetic groups with at least one genome sequenced with contigs of ⩾100 kb are shown with the help of the Tree of Life. The presence of identified *YBEY* genes is designated with green dots. *A YBEY homologue was detected only

in the *Planoprotostelium* genus and not in other amoebae. **YBEY was detected only in the *Mitosporidium* genus, the other microsporidia having lost the mitochondrial genome.

Figure S2. Mitochondrial localisation of YBEY. Immunostaining of transiently transfected HeLa and stably transfected HEK293T-REx cells expressing YBEY-3×FLAG. TOMM20 is used as mitochondrial marker. Scale bar, 10 µm.

Figure S3. Construction of CRISPR-mediated *YBEY* KO cell lines. (A) Genomic organisation of the *YBEY* locus. The annealing positions of gRNAs and the resulting deletions (Δ) are shown. **(B)** PCR validation of the introduced deletions. WT is the parental HEK293T-REx cell line. Three independent *YBEY*$^{+}$ cell lines were derived from WT as a result of the first (unsuccessful) round of CRISPR/Cas9 mutagenesis. Three *YBEY* KO cell lines were obtained from the corresponding *YBEY*$^{+}$ cell lines after a second transfection that resulted in disruption of all *YBEY* alleles. **(C)** The corresponding western blot confirms the lack of YBEY in *YBEY* KO cell lines.

Figure S4. *YBEY* KO results in selective deregulation of mitochondrial genes. (A) Relative mtDNA levels in *YBEY*$^{+}$ and *YBEY* KO cell lines, as assessed by qPCR. Means ± SEM for n = 9 independent cultures from three different cell lines for each genotype are shown; P-value, two-tailed Welch's test. **(B)** Snapshot of the mitochondrial *MT-CO1-MT-CO2-MT-ATP8/6* locus showing the downregulation of *MT-CO2* (and to a smaller extent *MT-CO1*) mRNAs in the absence of YBEY. "*8*" designates the small *MT-ATP8* ORF. tRNA genes, poorly covered by the employed RNA-Seq protocol, are not labelled for clarity. **(C,D)** smFISH analysis of *MT-CO1*, *MT-CYB* mRNAs, mt-tRNA^{Val} and 12S rRNA abundance in the *YBEY*$^{+}$ and *YBEY* KO cell lines. Mean total normalised RNA signal area ± SEM is shown in (D) for n = 20 (*YBEY*$^{+}$) and 10 (*YBEY* KO) frames (*MT-CO1*), n = 7 frames (*MT-CYB*), or n = 9 (*YBEY*$^{+}$) and 8 (*YBEY* KO) frames (mt-tRNA^{Val}), or n = 8 frames (12S rRNA), respectively; P-values, two-tailed Mann-Whitney test. **(E)** Probing total RNA for selected mitochondrial tRNAs by northern blotting reveals no significant differences between *YBEY*$^{+}$ and *YBEY* KO cells. 5S rRNA is used as loading control.

Figure S5. YBEY is not required for processing or polyadenylation of the various mitochondrial RNA species. (A) cRT-PCR analysis of the poly(A) tail length of 12S rRNA in *YBEY*$^{+}$ and *YBEY* KO cells. n = 77 plasmid clones from two independent *YBEY*$^{+}$ cell lines and n = 86 clones from two independent *YBEY* KO cell lines are represented by horizontal bars. **(B)** RT-qPCR analysis of the 16S rRNA precursor in *YBEY*$^{+}$ and *YBEY* KO cells. The 5' extension of the primary transcript into tRNA^{Val} and the 3' extension into tRNA$^{Leu(UUR)}$ were probed by qPCR across the respective junction. Means ± SEM for n = 3 are shown; P-values, two-tailed t-test. **(C)** RT-qPCR analysis of 4 mt-tRNA precursors with primers spanning 5' or 3' tRNA junctions. Means ± SEM for n = 3 independent *YBEY*$^{+}$ and *YBEY* KO cell lines are shown; P-values, two-tailed t-test. **(D)** cRT-PCR analysis of the 5'- and 3'-termini of the *MT-CO2* mRNA in *YBEY*$^{+}$ and *YBEY* KO cells. n = 45 (*YBEY*$^{+}$ cells) and 41 (*YBEY* KO) clones, represented by horizontal bars, are aligned with respect to the reference positions (a negative number means that the terminus is upstream, a positive – downstream, and "0" – at the expected reference position). **(E)** *YBEY*$^{+}$ and *YBEY* KO cells have similar proportions of correctly processed *MT-CO2* mRNA molecules. Proportions and 95% CIs are shown; based on the data in (D); P-value, Fisher's exact test. **(F)** cRT-PCR analysis of the poly(A) tail length of the *MT-CO2* mRNA in *YBEY*$^{+}$ and *YBEY* KO cells. n = 45 (*YBEY*$^{+}$ cells) and 41 (*YBEY* KO) clones, represented by horizontal bars. **(G)** cRT-PCR analysis of the positions of the 5'- and 3'-termini of 16S rRNA in *YBEY*$^{+}$ and *YBEY* KO cells. n = 100 clones from two independent *YBEY*$^{+}$ cell lines and n = 145 clones from three independent *YBEY* KO cell lines are aligned with respect to the reference positions, like in (D). Irregular truncation on 5'-ends of 16S rRNA are particularly frequent in *YBEY* KO cells. **(H)** Both *YBEY*$^{+}$ and *YBEY* KO cells have high proportions of correctly processed 16S rRNA molecules. However, 16S rRNA is frequently truncated in *YBEY* KO cells (appears as bars directed to the

right for the 5' termini or to the left for the 3' termini in panel (G)). Proportions and 95% CIs are shown; *P*-values, Fisher's exact test. **(I)** cRT-PCR analysis of the poly(A) tail length of 16S rRNA in *YBEY*$^{+}$ and *YBEY* KO cells. *n* = 100 clones from two independent *YBEY*$^{+}$ cell lines and *n* = 145 clones from three independent *YBEY* KO cell lines.

Figure S6. Recombinant human YBEY shows an endoribonuclease activity *in vitro*. (A) Typical Coomassie-stained gel of YBEY-His$_6$ purified on Ni-agarose under denaturing conditions (see Material and Methods for details). Fraction 7, eluted at 140 mM imidazole, was analysed by LC-MS/MS (all detected proteins are shown in the inset) and used in cleavage assays. **(B)** Predicted secondary structure of an RNA substrate composed of the 3' minor domain of mitochondrial 12S rRNA (black) and the adjacent tRNA^{Val} (red). The orange arrow shows the expected cleavage position to separate the 12S rRNA portion from tRNA^{Val}. The approximate position of the actual first major cleavage, as observed in the experiments in panels (C) and (D), is shown with the blue arrow. **(C)** The 12S rRNA 3'-minor domain-tRNA^{Val} substrate (50 ng) was incubated with increasing concentrations (200, 600, 1000, 2000 nM) of YBEY or the non-nucleolytic control protein uL18m purified under similar conditions, and the cleavage products were analysed by northern blotting with oligonucleotides annealing at the 5'- or the 3'-end of the substrate. The approximate position of the first major cleavage is shown with the blue arrow. The orange arrow shows the migration position of mature mt-tRNA^{Val}. **(D)** Saturating YBEY with Zn^{2+} does not change the cleavage pattern. The 12S rRNA 3'-minor domain-tRNA^{Val} substrate (50 ng) was incubated with increasing concentrations (200, 500, 800 nM) of YBEY expressed and purified in the presence of $ZnCl_2$, as described in Ref. 18, and the cleavage products were analysed as in (C). The approximate position of the first major cleavage is shown with the blue arrow. The orange arrow shows the migration position of mature mt-tRNA^{Val}. **(E)** The time-course of the YBEY-mediated cleavage of the precursor of the human mitochondrial tRNA^{Gln}.

Figure S7. Mutant variants of human YBEY successfully complement *YBEY* KO. (A) Mutations of conserved residues differentially affect the RNase activity of human YBEY (see also Figure 1A). The 12S rRNA 3'-minor domain-tRNA^{Val} substrate used in Supplementary Figure 6B-D was co-incubated with 500 nM purified YBEY proteins and the cleavage products were analysed by northern blotting. **(B)** WT and mutant YBEY versions successfully restore 12S rRNA levels in *YBEY* KO cells. Shown are means ± SEM for normalised 12S rRNA levels measured by RT-qPCR from *n* = 3 (WT) or *n* = 6 (*YBEY* KO cell lines) independent replicates; *P*-values, two-tailed Welch's test (all comparisons with the uncomplemented *YBEY* KO cells). **(C)** Metabolic [^{35}S]-methionine labelling of mitochondrial translation products reveals that the translational shutdown phenotype of *YBEY* KO cells can be complemented by WT and mutant YBEY alike. Western blots for YBEY proteins and VDAC1 and actin as loading controls are shown. All lanes are cropped from the same membrane. **(D)** WT and mutant YBEY restore normal cellular respiration, as assessed by oxygen consumption rate and extracellular acidification measurements by the Seahorse Mito Stress test. Means ± SEM for *n* = 7 (*YBEY*$^{+}$ and *YBEY* KO + R55A cell lines), or *n* = 8 (uncomplemented *YBEY* KO and *YBEY* KO + H128A), or *n* = 6 (*YBEY* KO + WT YBEY) are shown; *P*-values, two-tailed Welch's test.

Figure S8. YBEY forms a stable stoichiometric complex with p32 *in vivo*. (A) Cross-coimmunoprecipitation assays from HEK293T-REx cells that express in either combination YBEY-3×FLAG, p32-HA or untagged proteins with either FLAG- or HA-directed antibodies reveals specific reciprocal copurification of YBEY-3×FLAG and p32-HA proteins. Western blots probed for YBEY-3×FLAG, p32-HA and two non-interacting mitochondrial proteins, mL38 and PNPase, are shown. **(B)** Immunoprecipitation of endogenous p32 from the HEK293T-REx mitochondrial lysate specifically enriches endogenous YBEY, as compared to a control immunoprecipitation from the same lysate with an anti-FLAG antibody. Western blot analysis of lysate (L), flow-through (FT), wash (W) and

immunoprecipitate (IP) fractions is shown. **(C)** Analysis of the YBEY:p32 stoichiometry based on *n* = 6 and 4 coimmunoprecipitation experiments using YBEY and p32 (both endogenous and tagged) as a bait, respectively. Levels of copurified YBEY and p32 were measured by LC-MS/MS and spectral abundance factors for each protein were used to infer their stoichiometry. Medians, interquartile ranges and data ranges are shown.

Figure S9. YBEY forms a stable stoichiometric complex with p32 in a heterologous system (*E. coli*). (A) Purification of YBEY-His_6 and tagless p32, or both proteins together from native *E. coli* lysates on Ni-agarose columns. Coomassie-stained gels of total lysates (TL), soluble (Sol) and insoluble (Ins) fractions, flow-throughs (FT), washes (W), stepwise elutions with increasing concentrations of imidazole and the beads after the last elution are shown. Only the co-expression of YBEY-His_6 with p32 results in a partial solubilisation of the former and specific (at >100 nM imidazole) elution of both proteins. The molecular weight markers are the same as in Figure 5E. **(B)** Analysis of YBEY sedimentation in a high-resolution 10-40% glycerol gradient of the total cell lysate of HEK293T-REx cells. Western blots of YBEY, a small monomeric protein (mtIF3), mitochondrial SSU (mS35) and LSU (uL4m, uL24m, mL38) proteins are shown for comparison. **(C)** Control purification of tagless uS11m from the native *E. coli* lysate confirms that the protein is not retained on the Ni-agarose beads. Designations are the same as in (A).

Figure S10. *YBEY* KO does not change significantly the *MRPS11* mRNA level. Means ± SEM for *n* = 3 independent cell lines are shown; *P*-value, two-tailed t-test.

Figure S11. Isoelectric point distributions of YBEY proteins from various clades. Shown are proteobacteria (ancestors of mitochondria), cyanobacteria (ancestors of plastids), major photosynthetic (green) and non-photosynthetic (red) eukaryal phyla. Medians, interquartile ranges, 5th and 95th percentiles and kernel density distributions are provided.

Supplementary Figure 1

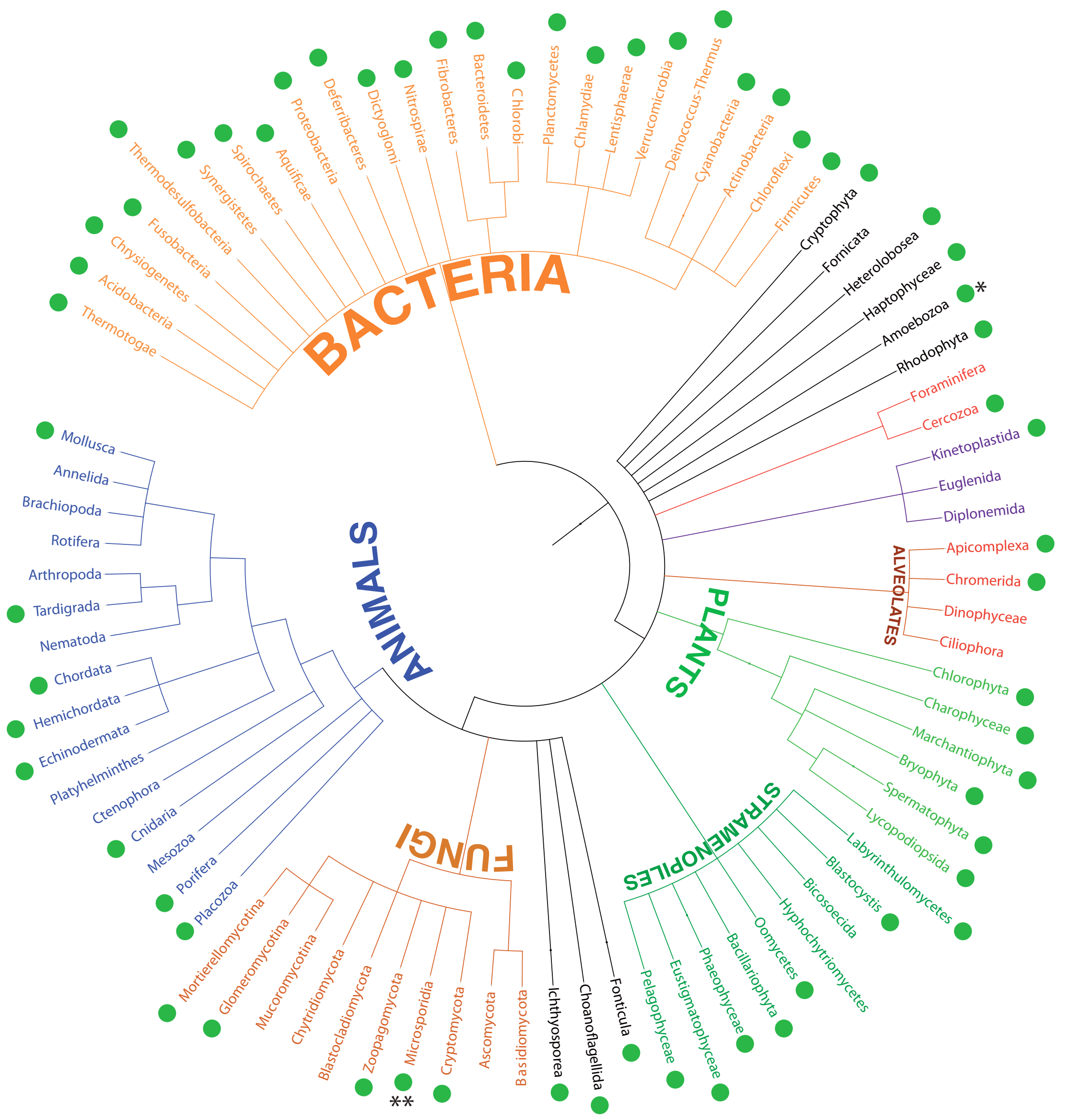

Supplementary Figure 2

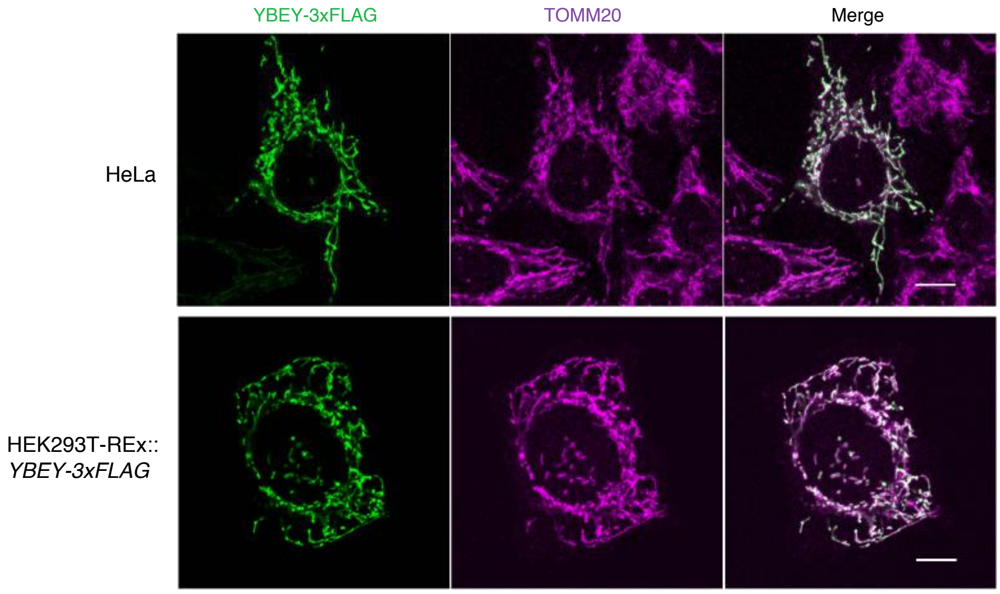

Supplementary Figure 3

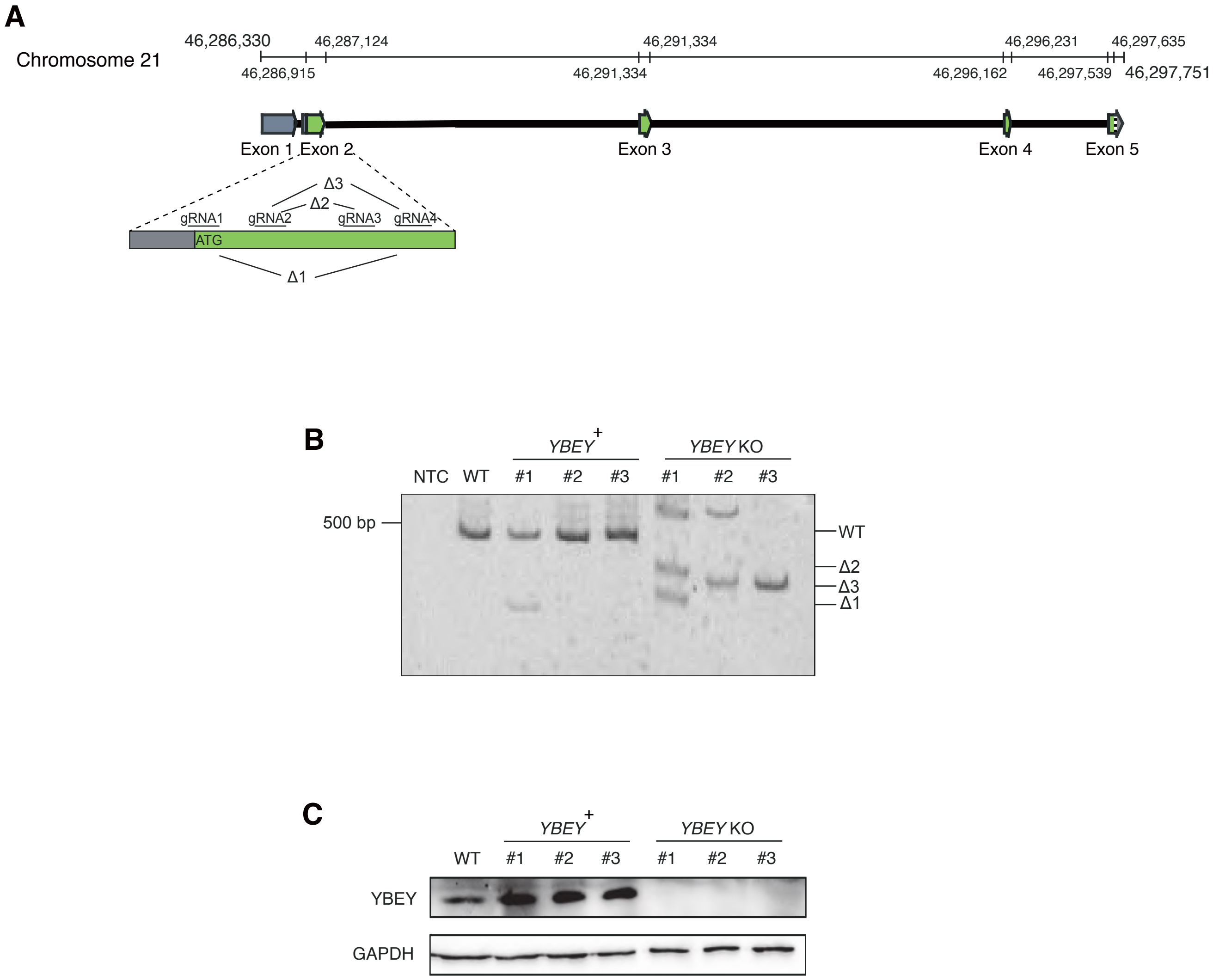

Supplementary Figure 4

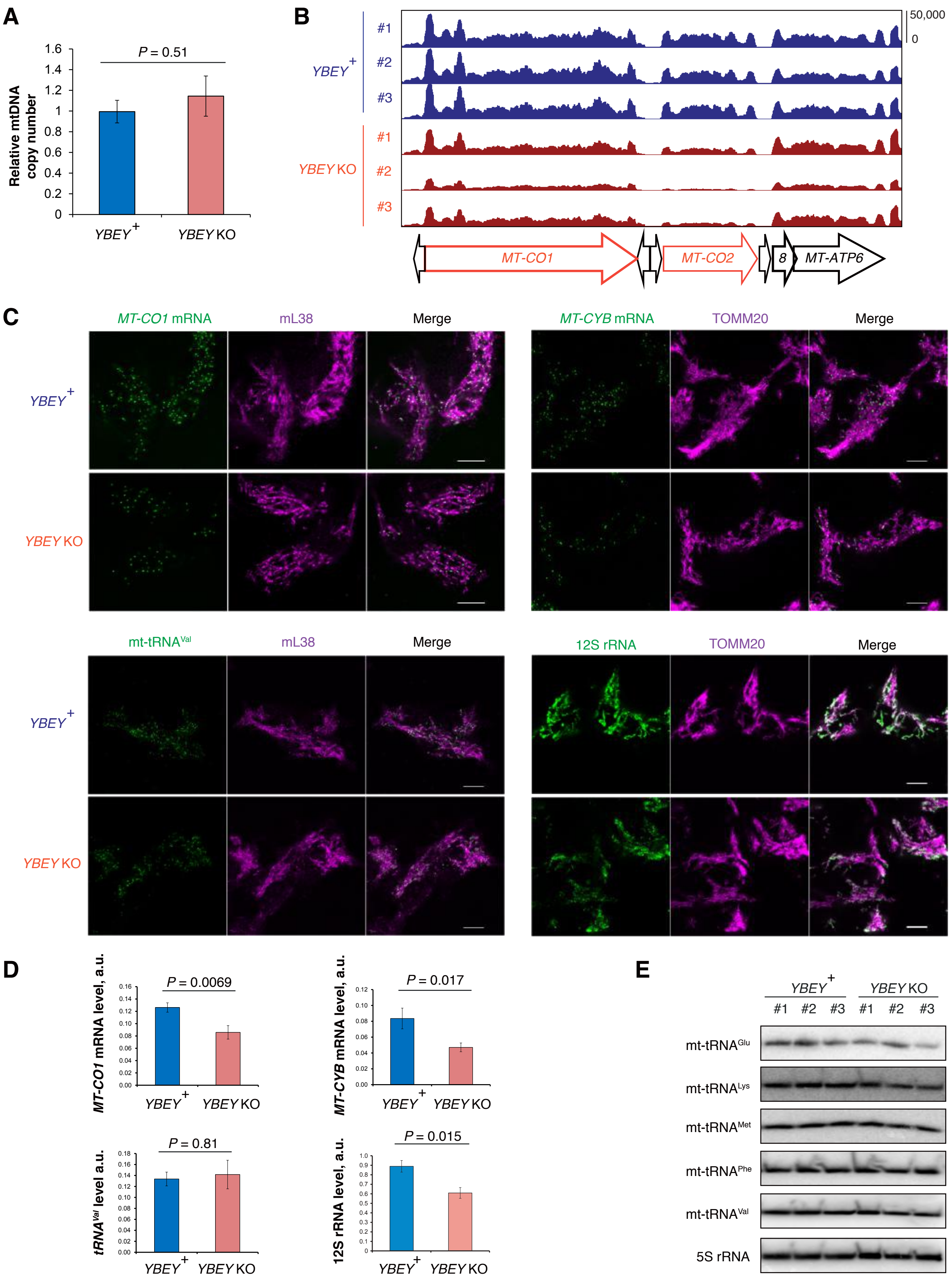

Supplementary Figure 5

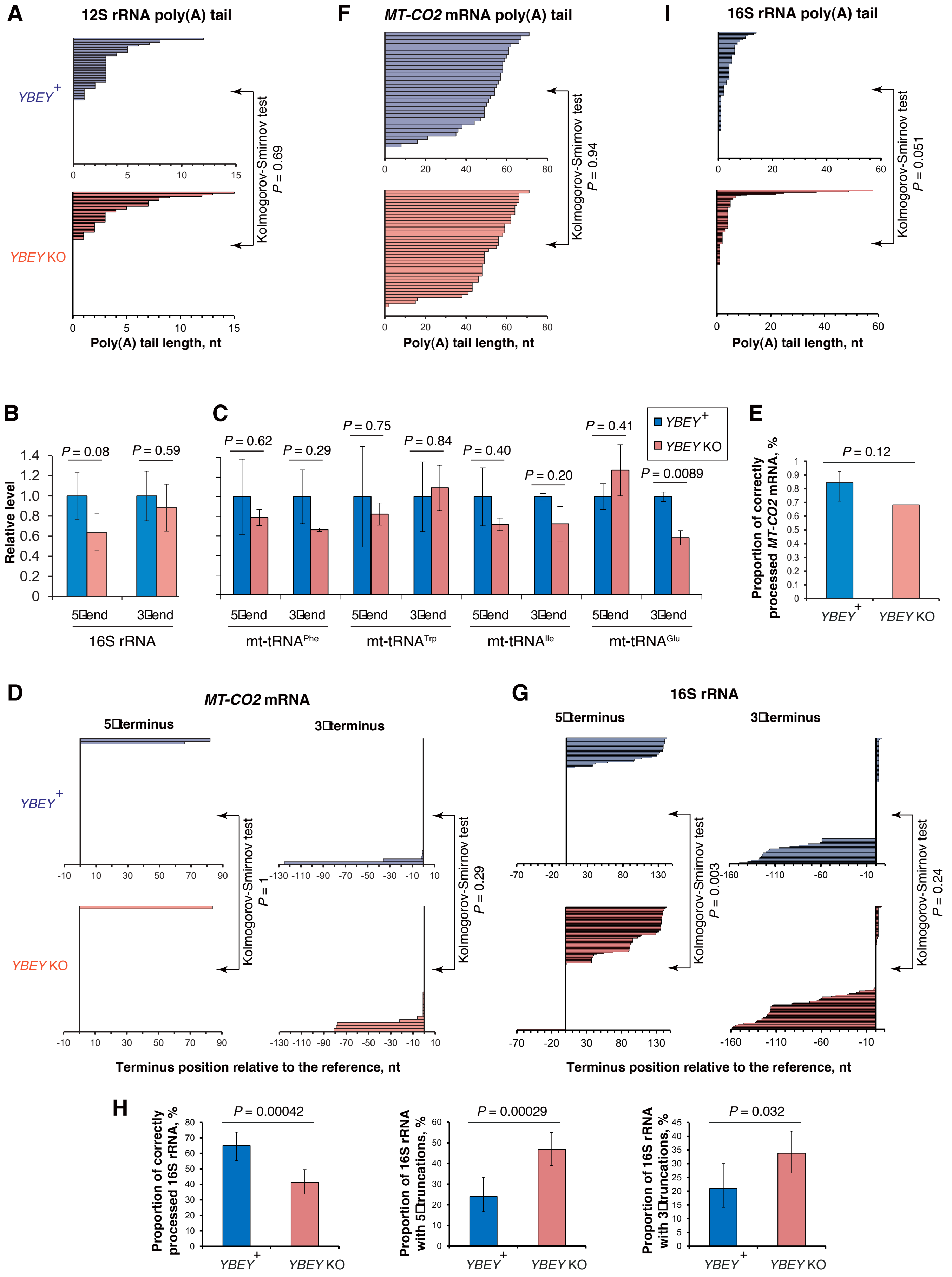

Supplementary Figure 6

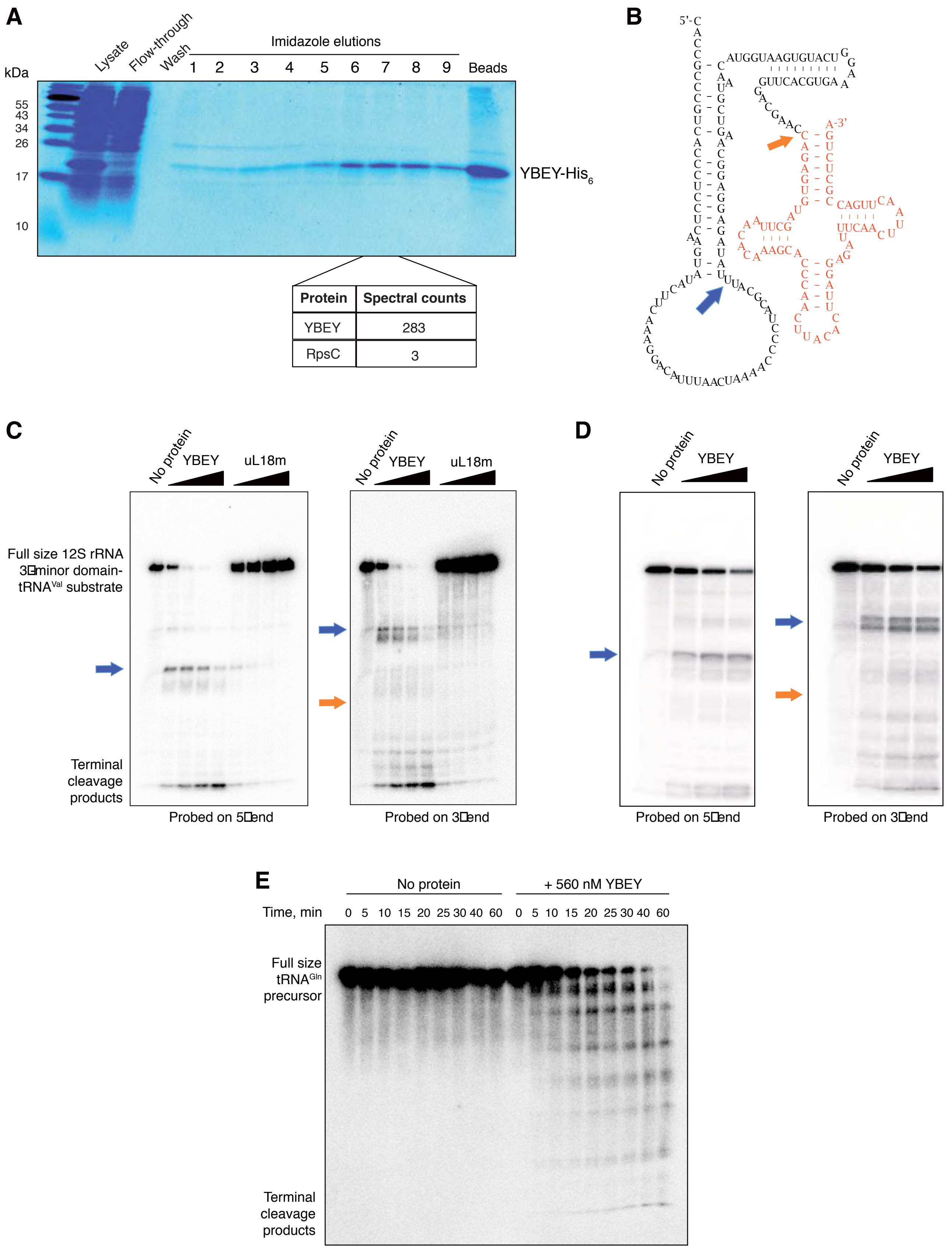

Supplementary Figure 7

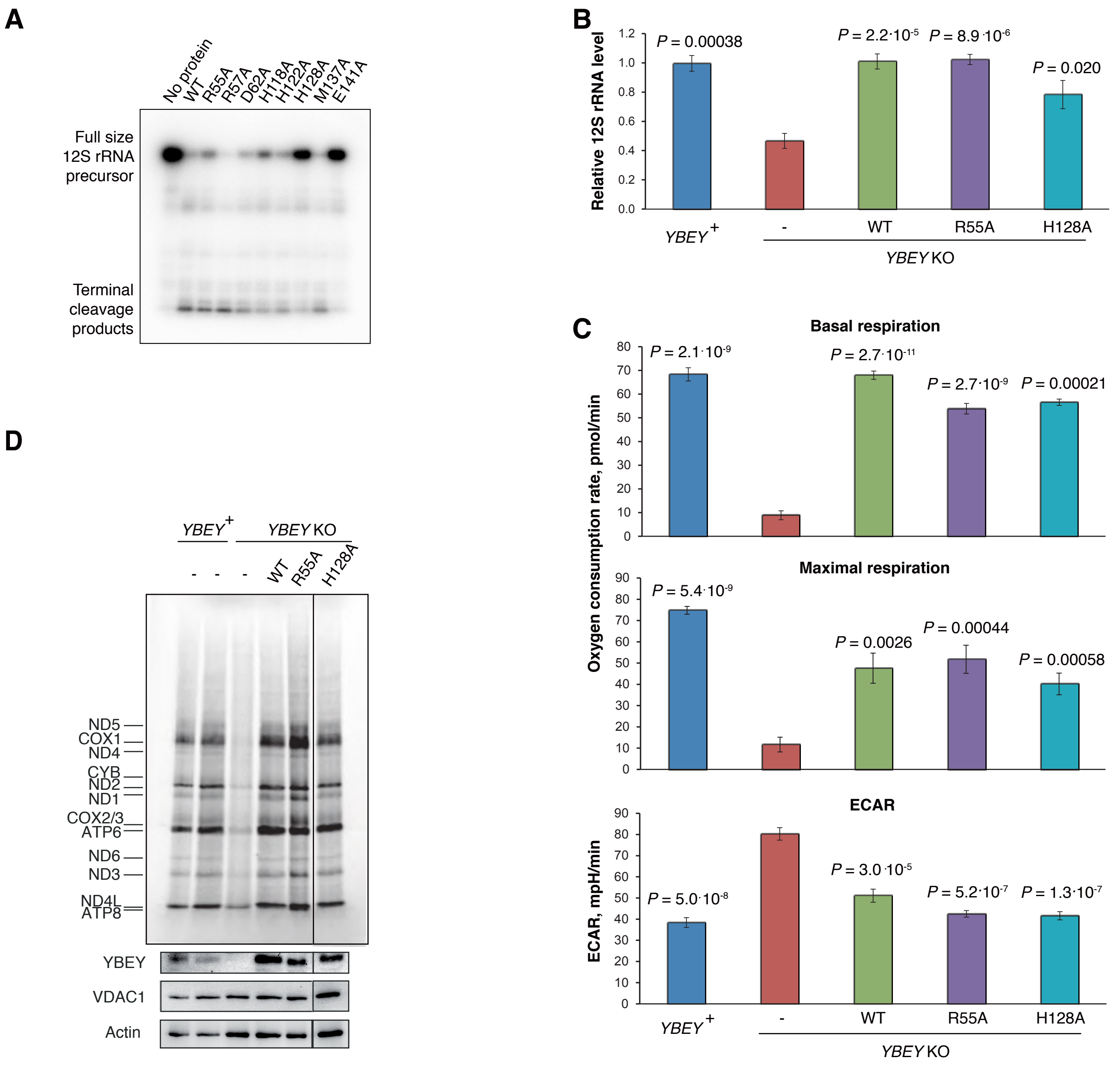

Supplementary Figure 8

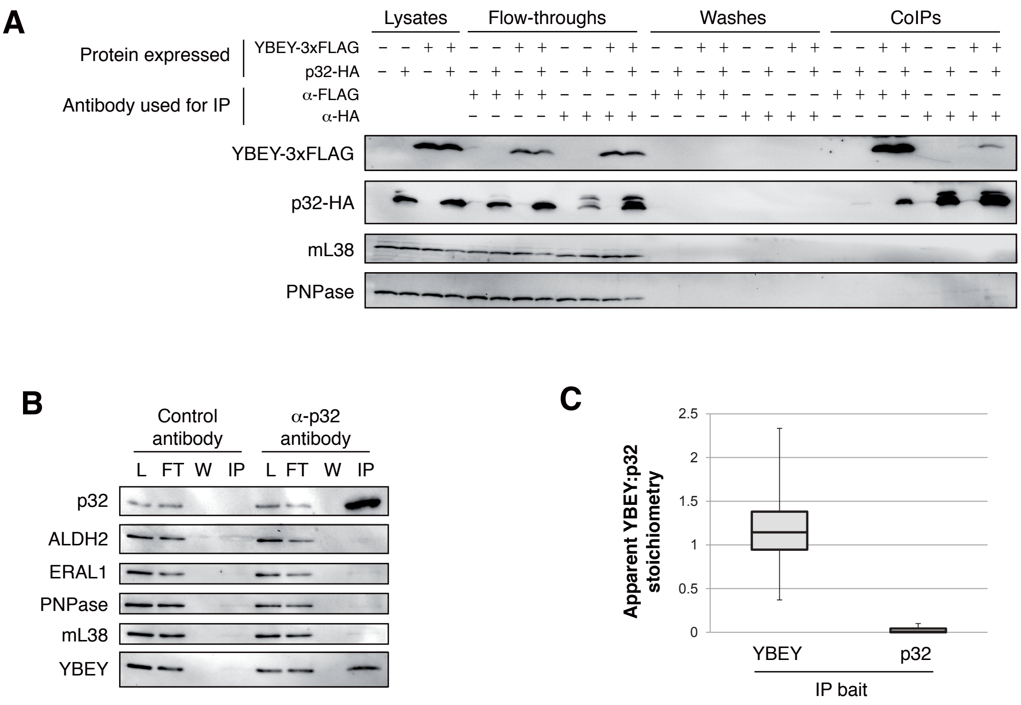

Supplementary Figure 9

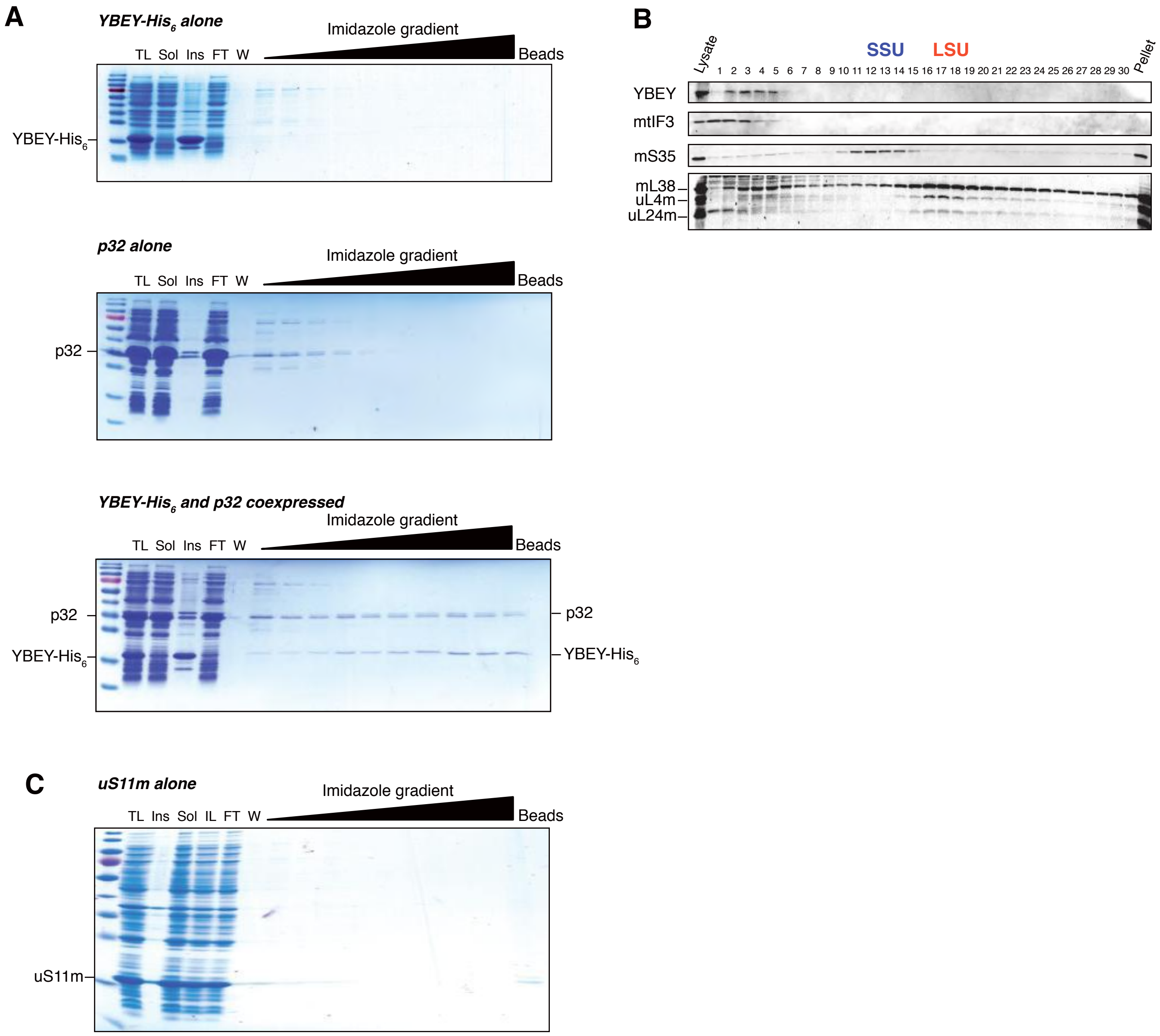

Supplementary Figure 10

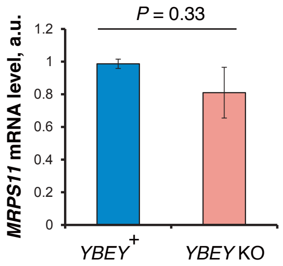

Supplementary Figure 11

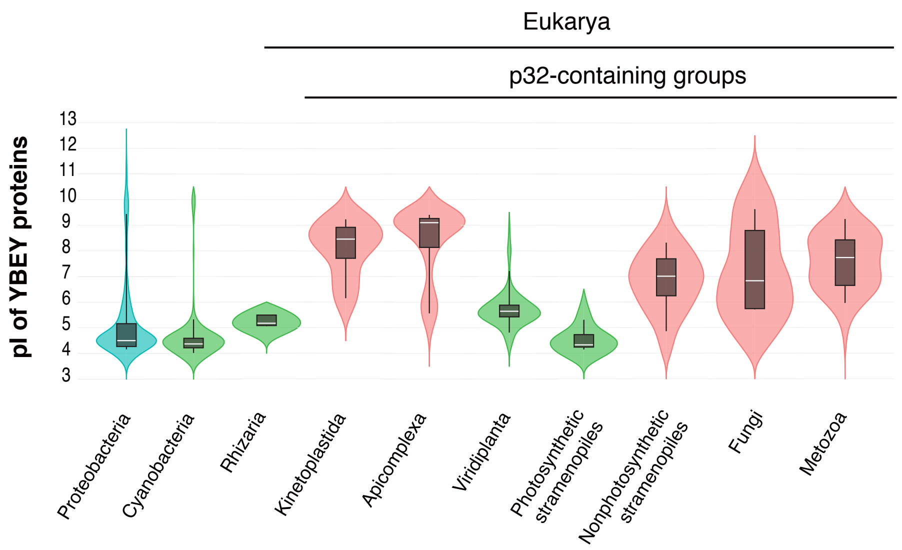

Supplement: gkaa148_Supplemental_Files [file gkaa148_supplemental_files.zip › Supplementary Data_revised_v04-min.pdf]
